# Pharmacokinetic Profile of Incremental Oral Doses of Dietary Nitrate in Young and Older Adults: A Crossover Randomized Clinical Trial

**DOI:** 10.1093/jn/nxab354

**Published:** 2021-11-09

**Authors:** Tess E Capper, Mario Siervo, Tom Clifford, Guy Taylor, Wasim Iqbal, Daniel West, Emma J Stevenson

**Affiliations:** Human Nutrition Research Centre, Population Health Sciences Institute, Faculty of Medical Sciences, Newcastle University, Newcastle upon Tyne, United Kingdom; Centre for Public Health, Queen's University Belfast, Belfast, United Kingdom; Human Nutrition Research Centre, Population Health Sciences Institute, Faculty of Medical Sciences, Newcastle University, Newcastle upon Tyne, United Kingdom; School of Life Sciences, The University of Nottingham Medical School, Nottingham, United Kingdom; Human Nutrition Research Centre, Population Health Sciences Institute, Faculty of Medical Sciences, Newcastle University, Newcastle upon Tyne, United Kingdom; School of Sport, Exercise and Health Sciences, Loughborough University, Loughborough, United Kingdom; Human Nutrition Research Centre, Population Health Sciences Institute, Faculty of Medical Sciences, Newcastle University, Newcastle upon Tyne, United Kingdom; Human Nutrition Research Centre, Population Health Sciences Institute, Faculty of Medical Sciences, Newcastle University, Newcastle upon Tyne, United Kingdom; Human Nutrition Research Centre, Population Health Sciences Institute, Faculty of Medical Sciences, Newcastle University, Newcastle upon Tyne, United Kingdom; Human Nutrition Research Centre, Population Health Sciences Institute, Faculty of Medical Sciences, Newcastle University, Newcastle upon Tyne, United Kingdom

**Keywords:** beetroot, nitric oxide, bioavailability, nitrate, ageing

## Abstract

**Background:**

Dietary nitrate consumption can increase concentrations of nitrate and nitrite in blood, saliva, and urine. Whether the change in concentrations is influenced by age is currently unknown.

**Objectives:**

We aimed to measure changes in nitrate and nitrite concentrations in plasma, urine, and saliva and exhaled NO concentrations after single incremental doses of dietary nitrate in young and older healthy adults.

**Methods:**

Twelve young (18–35 y old) and 12 older (60–75 y old) healthy, nonsmoking participants consumed single doses of 100 g, 200 g, 300 g whole beetroot (BR) and 1000 mg potassium nitrate (positive control) ≥7 d apart in a crossover, randomized clinical trial. Plasma nitrate and nitrite concentrations and exhaled NO concentrations were measured over a 5-h period. Salivary nitrate and nitrite concentrations were measured over a 12-h period and urinary nitrate over a 24-h period. Time, intervention, age, and interaction effects were measured with repeated-measures ANOVAs.

**Results:**

Dose-dependent increases were seen in plasma, salivary, and urinary nitrate after BR ingestion (all *P ≤* 0.002) but there were no differences between age groups at baseline (all *P ≥* 0.56) or postintervention (all *P ≥* 0.12). Plasma nitrite concentrations were higher in young than older participants at baseline (*P* = 0.04) and after consumption of 200 g (*P* = 0.04; +25.7 nmol/L; 95% CI: 0.97, 50.3 nmol/L) and 300 g BR (*P* = 0.02; +50.3 nmol/L; 95% CI: 8.57, 92.1 nmol/L). Baseline fractional exhaled NO (FeNO) concentrations were higher in the younger group [*P* = 0.03; +8.60 parts per billion (ppb); 95% CI: 0.80, 16.3 ppb], and rose significantly over the 5-h period, peaking 5 h after KNO_3_ consumption (39.4 ± 4.5 ppb; *P* < 0.001); however, changes in FeNO were not influenced by age (*P* = 0.276).

**Conclusions:**

BR is a source of bioavailable dietary nitrate in both young and older adults and can effectively raise nitrite and nitrate concentrations. Lower plasma nitrite and FeNO concentrations were found in older subjects, confirming the impact of ageing on NO bioavailability across different systems.

This trial was registered at www.isrctn.com as ISRCTN86706442.

## Introduction

Dietary patterns high in fruit and vegetables have been linked to reduced risk of diabetes and cardiovascular disease (1), but the identification of the key nutrients underpinning their beneficial effects remains elusive. Dietary nitrate (NO_3_^−^) content in green leafy and root vegetables [i.e., lettuce, cabbage, rocket, beetroot (BR), turnip] is considerable and, therefore, NO_3_^−^ has been considered as one of the putative nutrients closely linked to the cardioprotective effects of healthier dietary patterns such as the Mediterranean or Dietary Approaches to Stop Hypertension (DASH) dietary patterns (2). After ingestion, dietary NO_3_^−^ is reduced to nitrite (NO_2_^−^) by the activity of symbiotic oral bacteria with reductase capacity; NO_2_^−^ is further reduced to NO in the gastric acidic environment or in the peripheral vascular bed via the activity of specialized enzymes with reducing capacity (i.e., xanthine oxido-reductase, aldehyde dehydrogenase). The NO produced then has a positive impact on the vascular system through the control of vascular tone and endothelial integrity (3). This pathway is, therefore, amenable to targeted high-nitrate nutritional interventions, such as BR, aimed at preventing and/or treating conditions associated with a reduced NO production such as hypertension, diabetes, hypercholesterolemia, or chronic heart failure.

Dietary NO_3_^−^ has been found to be almost 100% bioavailable after the consumption of BR juice (4) and whole BR (5) in young, healthy individuals. Bioavailability studies can accurately map the pharmacokinetic profile of nutrients after consumption to identify excretory pathways, bioconversion, and the time course of physiological effects; however, these studies are frequently carried out in young populations (5–8). The ageing process has been found to have an impact on NO availability through lower concentrations of the precursor and cofactors involved in endogenous NO production (9). Studies have also indicated a reduced effect of dietary nitrate supplementation on blood pressure and endothelial function in older adults compared with younger adults (10). The reduced effect could be related to age-related changes in the pharmacokinetic profile of dietary NO_3_^−^ and bioconversion to NO_2_^−^ by bacterial species, which may be altered in older populations and affecting synthesis of NO and its downstream vasodilatory properties. There is a lack of pharmacokinetic data on incremental doses of dietary NO_3_^−^ and whether the kinetic curves are affected by ageing; previous studies have been conducted in younger populations and using nitrate salts, BR juice, or BR gel (7, 8). These pivotal studies first described the key role played by kidneys in handling inorganic nitrate, as well as identifying the nitrate entero-salivary circulation and incomplete recovery of orally ingested nitrate. Data on the pharmacokinetic profile of food sources of NO_3_^−^, such as whole BR or green leafy vegetables, are currently lacking and information is extrapolated from the kinetic curves of NO_3_^−^, after acute ingestion of labeled or unlabeled nitrate salts. van Velzen et al. (5) explored the bioavailability of nitrate-rich vegetables (i.e., cooked spinach, cooked BR, raw lettuce) in healthy subjects but changes in NO_3_^−^ concentrations were only measured in blood samples over a 24-h period.

The aim of this study was to investigate whether there is an age-dependent response in the pharmacokinetic profiles of incremental oral doses of dietary NO_3_^−^ from cooked whole BR compared with a high dose of potassium nitrate (KNO_3_) used as a positive control. The study adopted a multicompartment approach to evaluate, in younger and older healthy adults, changes in NO_3_^−^ and NO_2_^−^ concentrations in plasma (over a 5-h period postsupplementation), saliva samples (over a 12-h period postsupplementation), and urine samples (over a 24-h period postsupplementation) and measurement of NO in exhaled breath samples (over a 5-h period postsupplementation).

## Methods

### Subjects

Twenty-four nonsmoking, nonvegetarian young and older adults with no comorbidities were recruited; 12 participants aged 18–35 y  and 12 participants aged 60–75 y (see **Table 1** for physical characteristics). The **Supplemental Methods** provide exclusion criteria. Participants provided informed written consent before participating and the study (ISRCTN 86706442) was approved by the Cambridge Central Research Ethics Committee (16/EE/0376).

### Sample size calculation

The primary outcome measure was a difference in plasma bioavailability between these 2 forms of nitrate over a 5-h period. The 2 different forms of nitrate are the beetroot, and the positive control, the potassium nitrate. The sample size calculation was based on preliminary data from a previous, similar trial testing the acute effects of inorganic nitrate supplementation compared with placebo in young and older healthy individuals (11). The study found overall greater concentrations of plasma nitrate in younger than in older individuals over a 3-h sampling period and the estimated effect size *f* (partial η^2^) for a within-between repeated-measure ANOVA was 0.03 (0.175). Using these parameters in the same ANOVA model, we calculated that 12 participants/group (90/8 = 11.25) would be needed to detect a significant difference between doses and age groups with a power of 80% and *P* < 0.05. Sample size calculations were performed using G*Power 3.1 for Windows (Heinrich-Heine-Universität Düsseldorf, Germany).

### Study design

This study was a randomized, open-label, crossover clinical trial that took place in the Newcastle National Institute for Health Research Clinical Research Facility (CRF) of the Royal Victoria Infirmary, Newcastle upon Tyne. After telephone screening for inclusion criteria, participants attended a screening visit where blood pressure and BMI were measured for eligibility. Eligible participants attended the research facility on 4 separate occasions and they were allocated to receive, in a random order, the following interventions: *1*) 100 g whole cooked BR; *2*) 200 g whole cooked BR; *3*) 300 g whole cooked BR; and *4*) 200 mL KNO_3_ solution. The washout period was ≥7 d to allow for the complete return of NO_3_^−^ and NO_2_^−^ to presupplementation concentrations between visits. Randomization was conducted by an independent third party and the order of the interventions was determined using an online randomization software (www.randomizer.org). Specifically, the randomization process included the generation of consecutive, independent sets of numbers containing 4 numbers/set which ranged from 1 to 4. Each number was a priori allocated to a specific intervention. Each participant was consecutively allocated to a specific randomization sequence if they were deemed eligible to be enrolled in the study after the screening visit.

**FIGURE 1:**
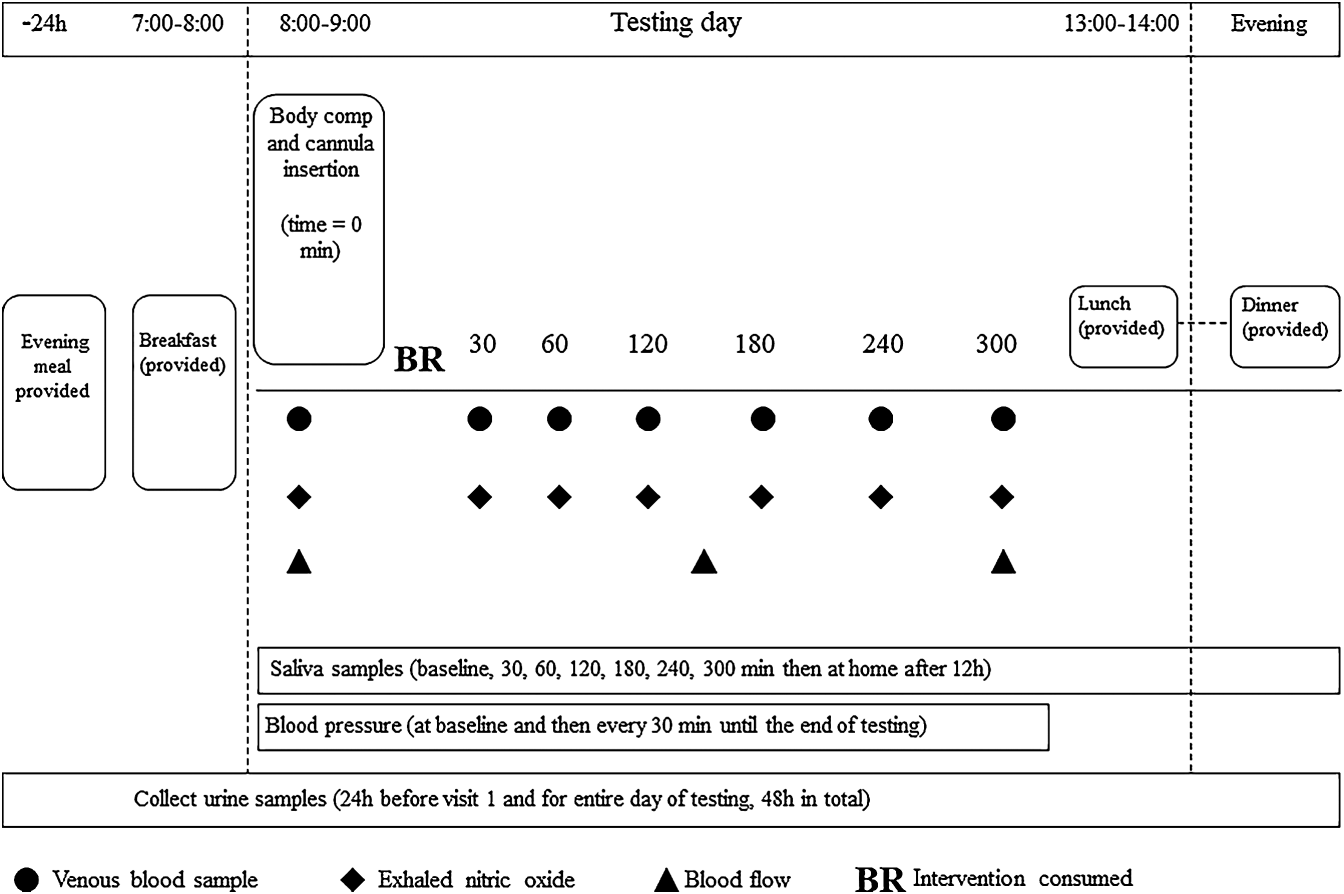
Testing day protocol.

Visits were conducted between 08:00 and 14:00 after a ≥10-h fast (water permitted). At the beginning of each visit, a cannula was fitted and participants provided baseline blood and saliva samples followed by baseline measurement of fractional exhaled nitric oxide (FeNO). Participants consumed the allocated BR (precooked and sliced) and KNO_3_ solution (consumed from the bottle as prepared) within 15 min and they were then provided with a standardized breakfast of cereal with milk, white toast, and a natural yoghurt pot to consume within 15 min. The meal contained ∼450 kcal with 62% of energy coming from carbohydrate, 16% from protein, and 22% from fat. Breakfast items were chosen from a list of low-NO_3_^−^ foods described by Wang et al. (12) and the NO_3_^−^ content of the meal was ∼2.8 mg. Participants were given nitrate-free bottled water (Buxton Water, Nestlé) for the 24-h testing day, and the volume consumed at the first visit was noted and replicated in subsequent visits. The intervention period began after the consumption of the intervention and lasted 5 h. As detailed in **Figure 1**, venous blood and saliva samples and FeNO were measured at times 30, 60, 120, 180, 240, and 300 min, and urine samples were taken at 0–3 h and 3–6 h. Urine and saliva samples were also measured at home at 6–12 h and 12–24 h and at 12 h, respectively.

### Treatments and dietary plans

The BR was purchased from a single grower (G's Fresh) as a precooked product in order to reduce variation in NO_3_^−^ content. The NO_3_^−^ content in BR was variable during the growing season and we were unable to purchase a single batch of the product due to the short shelf-life of the product. However, representative samples of BR from 3 batches were analyzed and averaged, and the interventions were, therefore, estimated to contain the following: 100 g BR, 272 mg NO_3_^−^; 200 g BR, 544 mg NO_3_^−^; and 300 g BR, 816 mg NO_3_^−^. The positive control contained 1000 mg KNO_3_, which was provided in a 200-mL sterile solution prepared by the Newcastle Royal Victoria Infirmary pharmacy. Participants were required to follow a low-NO_3_^−^ diet in the 2 d before each visit, as well as for the entirety of the testing day (24 h), as described previously (6, 13, 14). Compliance with the dietary plan was assessed with a self-reported 2-d diet diary. All participants were also asked to refrain from using antibacterial mouthwash and chewing gum throughout the study because of their potential influence on the conversion of NO_3_^−^ to NO_2_^−^ (15).

### Biological samples

Blood samples, saliva samples, and FeNO were collected before supplementation and at 30, 60, 120, 180, 240, and 300 min postsupplementation. A final saliva measurement was taken 12 h postsupplementation.

#### Blood sampling

Whole blood samples were collected via cannula into two 6-mL lithium heparin vacutainers (BD). One lithium heparin tube was centrifuged immediately at 5000 rpm (2795 × *g*; 4°C) for 3 min; the plasma was divided into aliquots in dark-colored Eppendorf tubes and frozen at −80°C to preserve the NO_2_^−^. These Eppendorf tubes were pretreated with a stop solution to prevent transition between compounds, as described by Nagababu and Rifkind (16). The remaining vacutainer was kept cool and processed within 30 min. The sample was then centrifuged at 3000 rpm (1006 × *g*) for 10 min at 4°C and the plasma was divided into aliquots and frozen at −80°C. The plasma samples were used to measure NO_3_^−^ and NO_2_^−^ concentrations.

#### Saliva and urine sampling

Stimulated saliva samples were collected by asking participants to chew a small cotton wool ball for ∼2 min; the cotton ball was then placed in the barrel of a sterile 20-mL syringe and the plunger was used to squeeze and collect the saliva sample into a 1-mL Eppendorf tube pretreated with 3.7 μL sodium hydroxide (NaOH, 1 M) solution (17). Participants also collected a sample at home 12 h postintervention.

Participants were asked to collect their 24-h total urine volume the day before each visit in a 3-L urine container pretreated with 5 mL of a 1-M NaOH solution. After each NO_3_^−^ dose, urinary NO_3_^−^ was measured over 4 time periods: 0–3 h (collected at the CRF), 3–6 h (collected at the CRF), 6–12 h (collected at home), and 12–24 h (collected at home). An aliquot of the samples in each collection period was deposited into a 30-mL container pretreated with 100 μL of 1 M NaOH solution. Saliva and urine samples were frozen at −20°C until analysis.

#### Breath FeNO

FeNO was measured using a handheld device (NIOX VERO, Circassia). Participants breathed steadily and consistently into the handheld sensor until a suitable measurement was taken, as signaled by the device.

### Plasma nitrate and nitrite analysis

Plasma NO_3_^−^ and NO_2_^−^ concentrations were measured at baseline, 60, 180, and 300 min. Plasma samples were first deproteinized before analysis using cold ethanol precipitation. The ozone-based chemiluminescence method was used to measure plasma and salivary NO_3_^−^ and NO_2_^−^ concentrations, and urinary NO_3_^−^ concentrations using the Sievers gas-phase chemiluminescence nitric oxide analyzer (NOA 280i, Analytix), which has been described elsewhere (18). The Supplemental Methods present a detailed description of the methodology for analysis of the BR samples.

### Statistical analysis

All statistical analyses were completed using IBM SPSS version 24.0 (SPSS Inc.). Summary data are presented as means ± SEMs with 95% CIs. Independent *t* tests were used to assess differences in baseline measures between the 2 age groups. Changes over time (0–5 h postintervention), intervention (100–300 g BR and 1000 mg KNO_3_), and time × intervention interaction effects were analyzed using a 3-factor mixed-model ANOVA with time and intervention as within-subjects factors and age as a between-subjects factor. Statistical analysis is presented for time, intervention, interaction, and age effects. Models were checked for sphericity using Mauchly's test and multivariate models were applied if these assumptions were violated. Where significances were found, a Bonferroni post hoc test was run. Statistical significance was set at *P <* 0.05.

## Results

**FIGURE 2:**
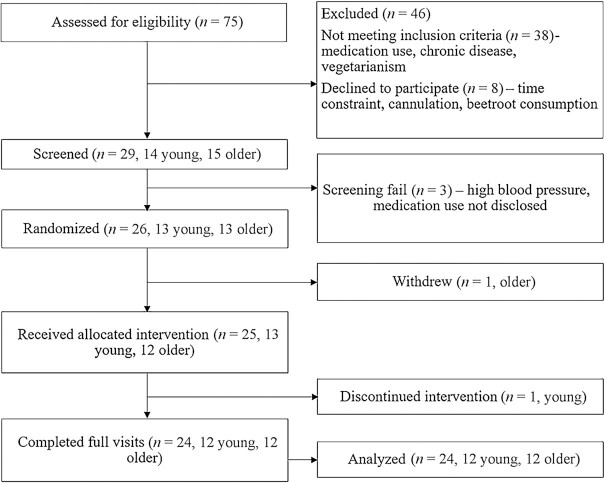
Recruitment flowchart.

**Figure 2** presents a flowchart of volunteer recruitment and Table 1 presents the baseline characteristics of the included participants. Age groups were matched for height and BMI but body weight was higher in the older group. The interventions were well tolerated and no adverse events were reported, although participants did describe beeturia and fecal discoloration after ingestion of the BR. All participants complied with the low-NO_3_^−^ diet according to the completed food diaries.

**TABLE 1:** Baseline characteristics of young and older participants^1^

	Young	Older	*P* value
*n*	12	12	
Gender, male/female	6/6	3/9	
Age, y	27.3 ± 1.09	64.3 ± 1.34	<0.001
Height, cm	172 ± 2.17	166 ± 2.46	0.07
Weight, kg	70.0 ± 1.30	66.4 ± 1.14	0.04
BMI, kg/m^2^	23.6 ± 0.30	24.2 ± 0.42	0.26
Waist circumference, cm	80.3 ± 0.69	85.5 ± 0.95	<0.001
Body fat, %	22.6 ± 1.30	30.8 ± 1.35	<0.001
SBP, mm Hg	115 ± 2.44	120 ± 3.50	0.27
DBP, mm Hg	72.6 ± 1.21	76.5 ± 2.03	0.11
Nitrate intake, mg/d	224 ± 21.1	253 ± 21.6	0.34
Physical activity, MET-min/wk	4220 ± 350.9	3460 ± 374.5	0.14
Plasma nitrate, μmol/L	28.5 ± 5.00	25.5 ± 3.68	0.63
Plasma nitrite, nmol/L	136 ± 6.27	114 ± 7.77	0.04
Salivary nitrate, mmol/L	0.85 ± 0.30	0.70 ± 0.14	0.65
Salivary nitrite, μmol/L	190 ± 32.0	162 ± 34.4	0.56
FeNO, ppb	30.7 ± 3.31	22.1 ± 2.04	0.03
Urinary nitrate concentration, mmol/L	0.64 ± 0.08	0.57 ± 0.06	0.47

1 Values are means ± SEMs. DBP, diastolic blood pressure; FeNO, fractional exhaled nitric oxide; MET, metabolic equivalents; SBP, systolic blood pressure.

### Plasma nitrate and nitrite

**FIGURE 3:**
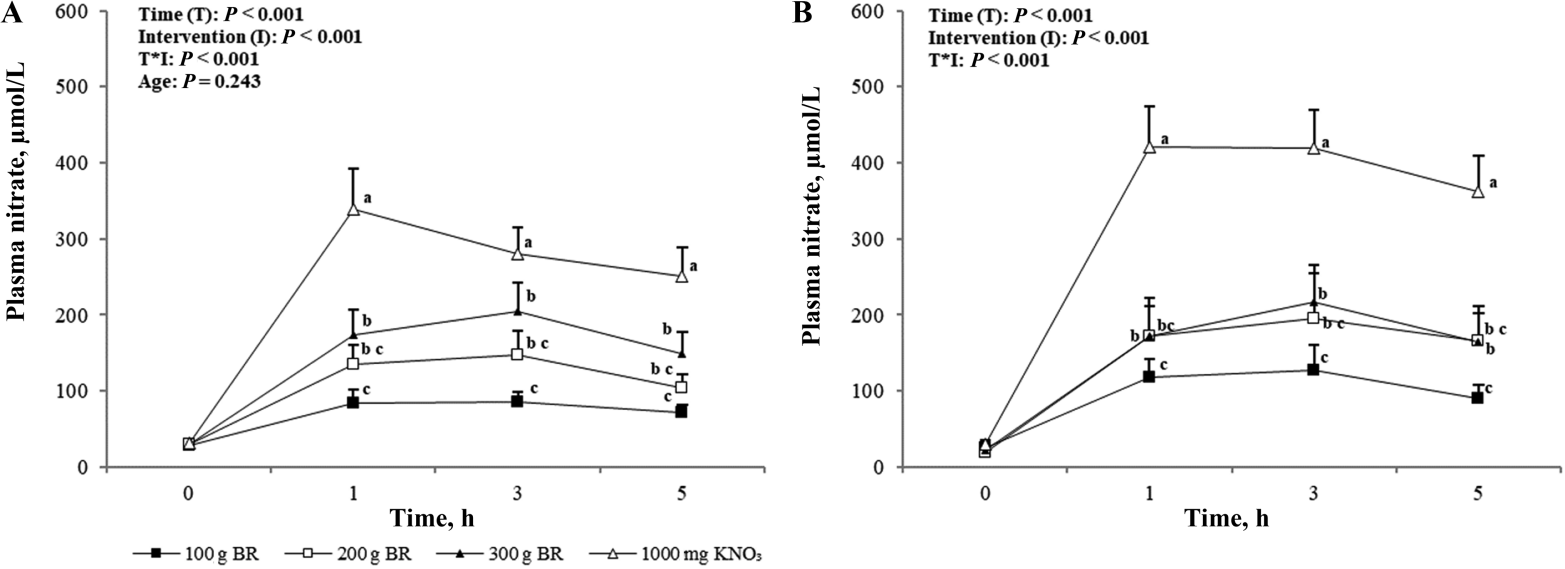
Mean ± SEM plasma nitrate concentrations in the young (A, *n* = 12) and older (B, *n* = 12) adults over 5 h after incremental doses of nitrate. Statistical analysis using 3-factor repeated-measures ANOVA (time × intervention × age). Labeled means at a time point without a matching letter differ, *P* < 0.05. Young and older age groups are presented separately to improve readability. BR, beetroot; KNO_3_, potassium nitrate.

Presupplementation, baseline plasma NO_3_^−^ concentrations were not different between age groups (*P* = 0.63) or trials (*P* = 0.46). After supplementation, there were significant effects of intervention, time, and their interaction on plasma NO_3_^−^ (all *P* < 0.001). There was no significant difference between age groups (*P* = 0.24). Plasma NO_3_^−^ concentration remained elevated above baseline at each time point over the 5-h intervention period (*P* < 0.001), and this occurred in all interventions (*P* < 0.001). Peak plasma NO_3_^−^ concentration occurred 3 h postadministration of BR after 100 g, 200 g, and 300 g BR, respectively, and 2-h postadministration of KNO_3_ (**Figure 3**).

**FIGURE 4:**
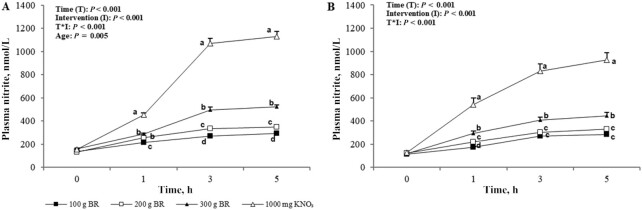
Mean ± SEM plasma nitrite concentrations in the young (A, *n* = 12) and older (B, *n* = 12) adults over 5 h after incremental doses of nitrate. Statistical analysis using 3-factor repeated-measures ANOVA (time × intervention × age). Labeled means at a time point without a common letter differ, *P* < 0.05. Young and older age groups are presented separately to improve readability. BR, beetroot; KNO_3_, potassium nitrate.

Baseline plasma NO_2_^−^ concentration was significantly higher in the young group than in the old group (*P* = 0.04; +21.5 nmol/L; 95% CI: 0.80, 42.2 nmol/L). There were significant effects of time, intervention, and their interaction on plasma NO_2_^−^ (*P* < 0.001); in addition, a significant difference between age groups was found (*P* = 0.005). Pairwise comparisons revealed dose-dependent increases in plasma NO_2_^−^ concentrations over time and with increasing NO_3_^−^ doses (*P* < 0.001) (**Figure 4**). Higher plasma NO_2_^−^ concentrations were found in the young group than in the older group after supplementation (+47.4 nmol/L; 95% CI: 16.1, 78.7 nmol/L). Specifically, post hoc analyses showed that plasma NO_2_^−^ concentrations in young participants were higher than in older participants after 200 g BR (*P* = 0.04; +25.7 nmol/L; 95% CI: 0.97, 50.4 nmol/L) and 300 g BR (*P* = 0.02; +50.3 nmol/L; 95% CI: 8.57, 92.1 nmol/L). There was a trend toward higher concentrations in the young after KNO_3_ ingestion (*P* = 0.06; +94.2 nmol/L; 95% CI: −4.53, 192 nmol/L). Over the intervention period, plasma NO_2_^−^ concentration in the young group was significantly higher than in the older group at 3 h (*P* = 0.001; +92.3 nmol/L; 95% CI: 42.5, 142 nmol/L) and 5 h (*P* = 0.006; +76.7 nmol/L; 95% CI: 24.4, 129 nmol/L). For both age groups, plasma NO_2_^−^ concentration peaked at 5 h postadministration for all NO_3_^−^ doses.

### Salivary nitrate and nitrite

**FIGURE 5:**
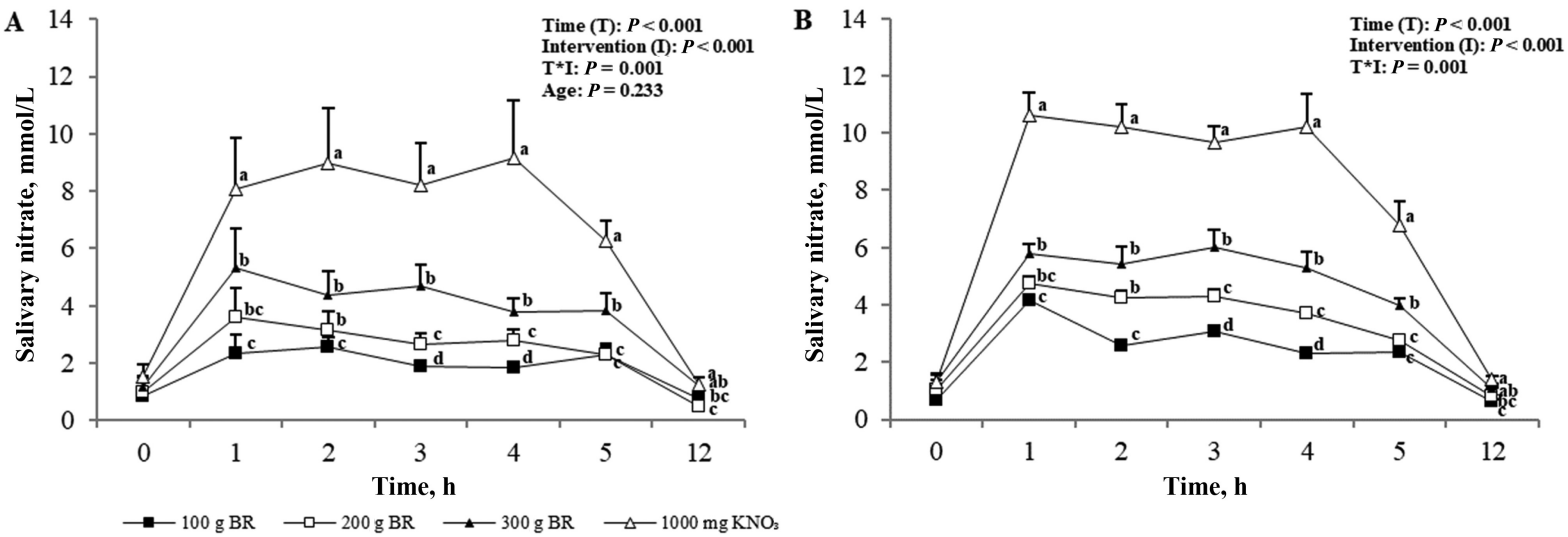
Mean ± SEM salivary nitrate concentrations in the young (A, *n* = 12) and older (B, *n* = 12) adults over 5 h after incremental doses of nitrate. Statistical analysis using 3-factor repeated-measures ANOVA (time × intervention × age). Labeled means at a time point without a common letter differ, *P* < 0.05. Young and older age groups are presented separately to improve readability. BR, beetroot; KNO_3_, potassium nitrate.

At baseline, there was no difference in salivary NO_3_^−^ concentration between age or intervention groups; in addition, changes in salivary NO_3_^−^ after supplementation did not differ between age groups (*P* = 0.23). Salivary NO_3_^−^ concentrations significantly increased in a dose-dependent manner (*P* = 0.002) and concentrations returned to baseline by 12 h in all intervention groups (**Figure 5**). Peak elevation in salivary NO_3_^−^ concentration occurred 1 h after the administration of the BR doses but a peak was instead reached at 4 h after the KNO_3_.

**FIGURE 6:**
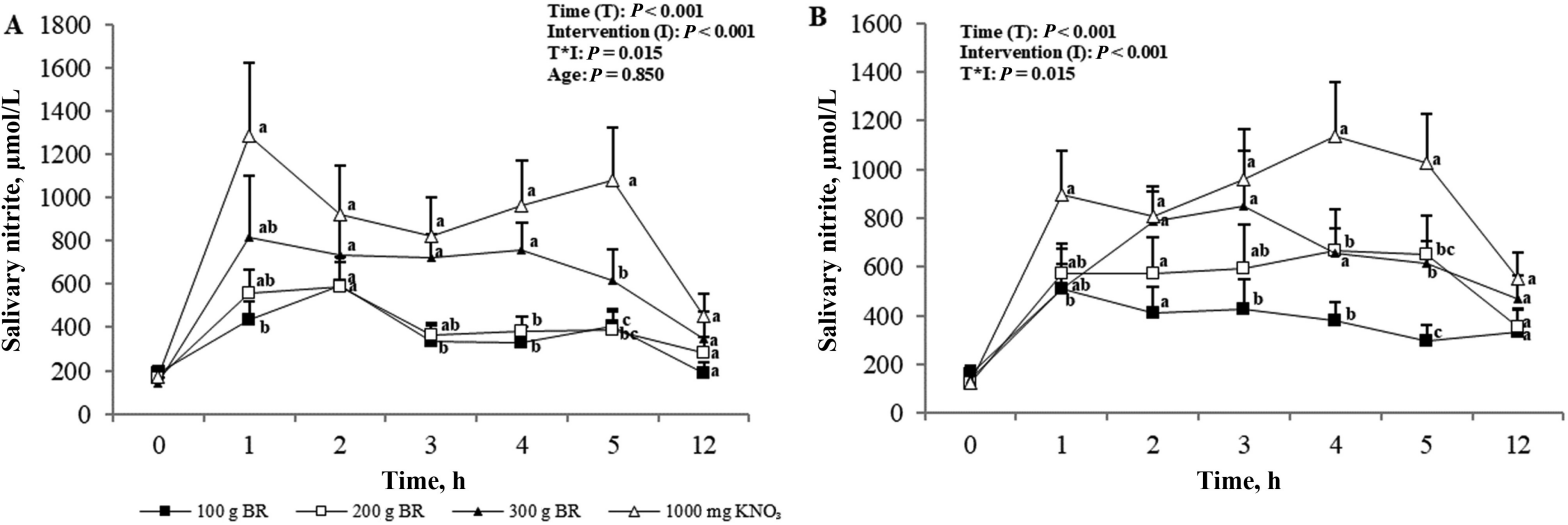
Mean ± SEM salivary nitrite concentrations in the young (A, *n* = 12) and older (B, *n* = 12) adults over 5 h after incremental doses of nitrate. Statistical analysis using 3-factor repeated-measures ANOVA (time × intervention × age). Labeled means at a time point without a common letter differ, *P* < 0.05. Young and older age groups are presented separately to improve readability. BR, beetroot; KNO_3_, potassium nitrate.

Baseline salivary NO_2_^−^ concentrations did not differ between age or intervention group. There was a significant effect of intervention and time (*P* < 0.001) as well as for their interaction (*P* = 0.02) on salivary NO_2_^−^ concentrations; however, there were no differences between age groups (*P* = 0.85). Salivary NO_2_^−^ concentrations did not significantly change from baseline over the 5 h after the administration of 100 g BR, whereas dose-dependent significant increases were observed with higher doses; greater changes were observed with the KNO_3_ intervention but concentrations returned to baseline in all groups after 12 h (**Figure 6**). Time of peak salivary NO_2_^−^ concentration also varied across NO_3_^−^ doses: the peak occurred at 2 h after 100 g and 200 g BR, 3 h after 300 g BR, and 1 h after KNO_3_ (Figure 6).

### Urinary nitrate

**FIGURE 7:**
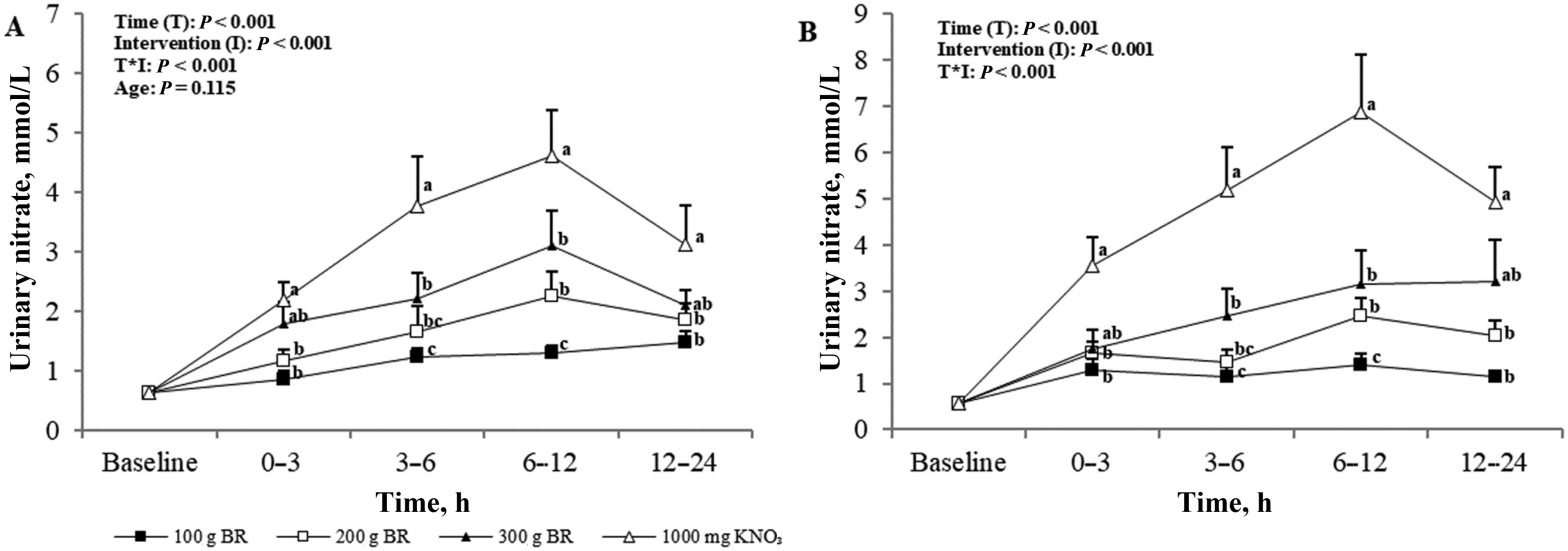
Mean ± SEM urinary nitrate concentrations in the young (A, *n* = 12) and older (B, *n* = 12) adults over 5 h after incremental doses of nitrate. Statistical analysis using 3-factor repeated-measures ANOVA (time × intervention × age). Labeled means at a time point without a common letter differ, *P* < 0.05. Young and older age groups are presented separately to improve readability. BR, beetroot; KNO_3_, potassium nitrate.

Baseline urinary NO_3_^−^ concentrations were not different between age (*P* = 0.56) or intervention (*P* = 0.34) groups. We observed significant effects of intervention, time, and their interaction on urinary NO_3_^−^ concentrations (*P* < 0.001) (**Figure 7**); changes in urinary NO_3_^−^ concentrations did not differ between age groups (*P* = 0.12). Urinary NO_3_^−^ concentrations significantly increased in a dose-dependent manner after the administration of the NO_3_^−^ doses (*P* < 0.001). Peak urinary NO_3_^−^ concentrations occurred 6–12 h postadministration for all NO_3_^−^ doses.

### FeNO

**FIGURE 8:**
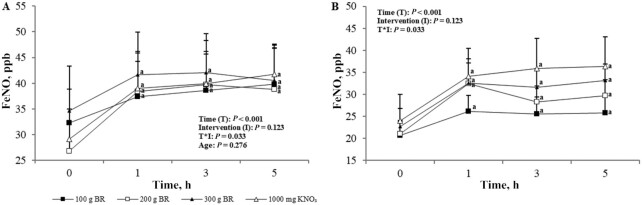
Mean ± SEM FeNO in the young (A, *n* = 12) and older (B, *n* = 12) adults over 5 h after incremental doses of nitrate. Statistical analysis using 3-factor repeated-measures ANOVA (time × intervention × age). Labeled means at a time point without a common letter differ, *P* < 0.05. Young and older age groups are presented separately to improve readability. BR, beetroot; FeNO, fractional exhaled nitric oxide; KNO_3_, potassium nitrate.

Baseline FeNO concentrations were significantly higher in the young than in the older group (*P* = 0.03). After supplementation, there was a significant effect of time (*P* < 0.001) but not age (*P* = 0.28) and intervention (*P* = 0.12) on changes in FeNO concentrations; the interaction between intervention and time was significant (*P* = 0.03). Changes in FeNO concentrations were significantly higher than baseline over the 5-h assessment period (*P* < 0.001) (**Figure 8**). Time of peak FeNO concentrations varied across NO_3_^−^ doses; the peak occurred at 5 h after 100 g BR and KNO_3_, 3 h after 200 g BR, and 3 h after 300 g BR (Figure 8).

## Discussion

As far as we know, this is the first study to investigate dose-dependent pharmacokinetic profiles of NO_3_^−^ and NO_2_^−^ in both young and older healthy participants measured in plasma, saliva, urine, and exhaled breath samples. Results show that whereas plasma NO_3_^−^ concentrations were comparable across age groups at baseline and after consumption of NO_3_^−^ doses, age affected plasma NO_2_^−^ concentrations, as shown by higher baseline NO_2_^−^ concentrations in the young than in the older group and a significant influence of age on NO_2_^−^ concentrations after NO_3_^−^ consumption. These results suggest age-related differences in basal NO production and response to NO_3_^−^ consumption in the form of whole BR.

Efficient absorption of dietary NO_3_^−^ from whole BR in both the young and older groups was demonstrated through the significant increase in plasma NO_3_^−^ to comparable concentrations in the groups. Although the smallest amount of BR, 100 g, significantly raised NO_3_^−^ concentrations, a dose-dependent response could be seen after 200 g and 300 g BR with a 1.6- and 2.0-fold greater rise in plasma NO_3_^−^ concentrations, respectively, across both age groups. The peaks at ∼3 h after ingestion of all doses of BR and ∼2 h after KNO_3_ ingestion are thought to accurately reflect the pharmacokinetic profile of plasma NO_3_^−^, echoing previous studies using BR and BR juice (5–7). The difference in time to peak for KNO_3_ most likely reflects the body's ability to process a solution faster than NO_3_^−^ from a whole vegetable. Kapil et al. (19) and Wylie et al. (7) have previously demonstrated similar pharmacokinetic profiles of NO_3_^−^ after KNO_3_ and BR juice supplementation and these, together with results of the present study, demonstrate the bioavailability of NO_3_^−^ from NO_3_^−^ salts, BR juice, and now practical amounts of the whole vegetable in young and older participants.

The appearance of NO_3_^−^ in the urine within 3 h after ingestion further demonstrates the bioavailability of NO_3_^−^ from whole BR, peaking between 6 and 12 h, which is consistent with earlier evidence from Pannala et al. (20) on urinary nitrate in adults aged between 20 and 50 y after a high-nitrate meal. Contrary to the study by Pannala et al. (20), however, urinary NO_3_^−^ concentration remained elevated at 12–24 h postingestion in the present study, indicating that NO_3_^−^ had not been cleared within 24 h. Dietary NO_3_^−^ has a half-life of ∼8 h and 60% of ingested NO_3_^−^ has been found to be excreted in the urine within 48 h (21); a longer sampling period in the present study may therefore have identified the time when concentrations returned to baseline.

Age-related factors such as changes to the oral microbiome are postulated to affect the NO_3_^−^–NO_2_^−^–NO pathway (22). Indeed, despite similar plasma NO_3_^−^ concentrations across the interventions, plasma NO_2_^−^ concentrations, both at baseline and after supplementation, were higher in the young than in the older adults. As with plasma NO_3_^−^ concentration, however, there was a dose-dependent response in plasma NO_2_^−^ concentrations to increasing NO_3_^−^ amounts consumed. Previous work has found contrasting impacts of dietary NO_3_^−^ on plasma NO_3_^−^ and NO_2_^−^ concentrations in older adults. For example, Presley et al. (23) first demonstrated significantly raised plasma NO_3_^−^ and NO_2_^−^ concentrations in older adults after a high-NO_3_^−^ diet plus BR juice containing 769 mg nitrate. Conversely, Miller et al. (24) found that a high-nitrate diet providing a maximum of 77 mg nitrate in a single bolus was not sufficient to increase plasma NO_3_^−^ and NO_2_^−^ concentrations in older adults. These together imply that the response to dietary NO_3_^−^ is dose dependent. The lower baseline plasma NO_2_^−^ concentrations found in the older group in the present study may suggest an impairment in endogenous NO production and/or conversion of NO_3_^−^ into NO_2_^−^. Lower baseline NO concentrations (for which plasma NO_2_^−^ is a marker) in older adults have been shown by Ashor et al. (25) and diminished NO production is accepted to be a symptom of the ageing process (10, 26). The conversion of NO_3_^−^ to NO_2_^−^ occurs in the mouth and, therefore, plasma NO_2_^−^ concentrations are increased after swallowing; this suggests that age differences lie in the oral conversion of NO_3_^−^ to NO_2_^−^. The later peak at 5 h seen for plasma NO_2_^−^ concentration demonstrates the utilization of the salivary pathway in the breakdown of NO_3_^−^ to NO_2_^−^ after first reaching the blood (15, 27). Salivary measurement is a relatively new addition to the body of evidence on dietary NO_3_^−^ but it provides a simple noninvasive biomarker of NO production. Here we demonstrate comparable salivary NO_3_^−^ and NO_2_^−^ concentrations in young and older participants before and after NO_3_^−^ ingestion. This contradicts the aforementioned theory of impaired NO_3_^−^ to NO_2_^−^ breakdown in the older group. However, although not statistically different to the young group, both plasma and salivary NO_3_^−^ concentrations in the older group were consistently higher after NO_3_^−^ consumption. Percentage change in plasma NO_3_^−^ concentrations after increasing NO_3_^−^ doses (100g, 200g, 300g of BR and 1000mg of KNO_3_) was 150%, 253%, 393%, and 717% in the young and 251%, 753%, 650%, and 1099% in the older adults, respectively. Similarly, although varying more with dose, change in salivary NO_3_^−^ concentrations after increasing NO_3_^−^ doses was 170%, 133%, 224%, and 312% in the young and 237%, 161%, 202%, and 415% in the older group, respectively. This suggests heightened utilization of dietary NO_3_^−^ in older participants, potentially as a protective mechanism against a reduced ability to convert NO_3_^−^ to NO_2_^−^. Further research in this area is warranted.

It is evident that 100 g BR, i.e., a dose of 272 mg NO_3_^−^, is not sufficient to significantly raise salivary NO_2_^−^ concentrations, despite raising salivary NO_3_^−^. This suggests that the small dose of dietary NO_3_^−^ does not stimulate the NO_3_^−^-reducing bacteria located in the salivary glands to reduce salivary NO_3_^−^ to NO_2_^−^. In 1976, it was determined that salivary NO_2_^−^ concentration was highly dependent on NO_3_^−^ ingestion and there was a limit <54 mg in which salivary NO_3_^−^ and NO_2_^−^ concentrations did not change (28). Bacteria use NO_3_^−^ and NO_2_^−^ as final electron acceptors in respiration when they are available in the oral cavity (28), therefore, a low availability from small NO_3_^−^ doses may affect respiration pathways in the bacteria. Higher doses of NO_3_^−^, i.e., ≥200 g BR, increased salivary NO_2_^−^ concentration, which suggests that these amounts are able to stimulate NO_3_^−^ to NO_2_^−^ reduction in the saliva.

Plasma and salivary NO_2_^−^ concentrations are biomarkers for NO production, which has been found to maintain aspects of the vascular system such as blood pressure and vascular tone (3). Dejam et al. (29) found that plasma NO_2_^−^ concentrations ≥350 nmol increased forearm blood flow and this concentration was not reached by BR amounts <300 g in the older group and <200 g in the young group in the present study. However, 300 g BR was found to raise plasma NO_2_^−^ to physiologically beneficial concentrations in both the young and the older group, at least in relation to forearm blood flow. Kapil et al. (19) demonstrated a systolic blood pressure–lowering effect with NO_3_^−^ intakes of ∼341 mg from BR juice but not with 248 mg from KNO_3_. This NO_3_^−^ dose can be achieved through the consumption of 200 g BR but not by smaller amounts. However, plasma NO_2_^−^ concentration remained elevated above baseline at the 5-h time point after all doses of BR, which suggests that NO concentration would also remain elevated for a longer period. Furthermore, each of the BR amounts raised FeNO concentrations within 1 h of consumption and these concentrations remained elevated over the 5-h intervention period. There was no difference between the interventions, showing that a small amount of BR, providing 272 mg NO_3_^−^, has the ability to increase NO concentrations in exhaled air. Again, demonstrating a lower endogenous production of NO_2_^−^ and NO, baseline FeNO concentrations in the present study were lower in the older group than in the young group. This, however, contradicts findings of previous studies with comparable baseline FeNO concentrations in younger and older adults (30, 31). In a study on adults aged 65–79 y using the same FeNO measurement device as the present study, Coggan et al. (30) demonstrated an increase in FeNO concentrations from ∼20 parts per billion (ppb) at baseline to between 40 and 50 ppb after consumption of 13.4 mmol NO_3_^−^. We found similar baseline FeNO concentrations in our older group of 22.1 ppb but a significantly higher baseline FeNO in the young group of 30.7 ppb. The relatively small sample size of older adults of 12, and consequently large interindividual variability, in both this study and Coggan et al. (30), may affect the significance of results. NO can be formed from NO_2_^−^ in the oral cavity and stomach (32) and elevated FeNO concentrations after an NO_3_^−^ load reflect utilization of this process.

This study has many strengths: the stringent screening process ensured that disease states or medications that may have confounded the effects of the interventions on the NO_3_^−^–NO_2_^−^–NO cycle were screened out; the addition of salivary NO_3_^−^ and NO_2_^−^ and urinary NO_3_^−^ analysis advances the body of research on the pharmacokinetics of dietary NO_3_^−^ from whole BR in an older population; and analysis was completed using ozone-based chemiluminescence, which is acknowledged as the gold-standard method and the most sensitive technique available for the measurement of NO and its metabolites (33). Nevertheless, this study is not without its limitations. The small sample size limits the application of this study and the conclusions that can be drawn from it. However, the crossover design and inclusion of both a young and an older participant group of equal size allowed for direct comparison and assessment of the impact of age on the dietary NO_3_^−^ pathway. Purchasing BR on a per-participant basis meant that the NO_3_^−^ content of the BR could have varied across participants. To counteract this, the BR purchased came from the same grower and all trials were conducted within as small a timeframe as possible to reduce the impact of NO_3_^−^ variability. Presenting the participants with BR meant that the trials were not blinded and measurements may have been unconsciously influenced by the allocation to the interventions.

Despite the limitations, data presented in this study provide new information on the bioavailability of whole BR in both young and older participants and highlight age-related differences in basal NO production and in utilization of dietary NO_3_^−^ from whole BR. Whole BR was a source of bioavailable NO_3_^−^ for both young and older participants. More research is warranted into the production of both NO_2_^−^ from dietary NO_3_^−^ and endogenous NO into older age, because it is evident that there is a reduced efficiency in one or the other of these pathways. Subsequent research should focus on whether whole BR, in practical amounts, has effects on physiological outcomes and whether the effects are modified by age.

## Supplementary Material

nxab354_Supplemental_File

## Notes

Supported as part of a doctoral degree that received financial support from G's Fresh Ltd (to EJS). The funders had no role in the study design, preparation, analysis, or writing of the manuscript.

Author disclosures: The authors report no conflicts of interest.

Supplemental Methods are available from the “Supplementary data” link in the online posting of the article and from the same link in the online table of contents at https://academic.oup.com/jn/.

Abbreviations used: BR, beetroot; CRF, Clinical Research Facility; FeNO, fractional exhaled nitric oxide; ppb, parts per billion.
