# Supplementary material for: Pharmacokinetic Profile of Incremental Oral Doses of Dietary Nitrate in Young and Older Adults: A Crossover Randomized Clinical Trial
Source: J Nutr. 2021 Nov 9;152(1):130–9. doi: 10.1093/jn/nxab354 (PMC8754575; doi:10.1093/jn/nxab354)
Supplement: nxab354_Supplemental_File [file nxab354_supplemental_file.docx]

**Supplementary Methods**

*Exclusion criteria*

Participants were excluded if they consumed >30 units alcohol per week, had a BMI >30kg/m^2^, blood pressure >140/90mmHg, history of repetitive gastric reflux or intolerance or allergy to beetroot, or were taking the following medications: corticosteroids, sildenafil, aspirin, non-steroidal anti-inflammatory drugs (NSAIDs), anti-hypertensives (Ca^2+^ channel blockers, ACE inhibitors), diuretics, beta-blockers, antacids, anticoagulants, nitrate-derived agents, and anti-cholinergics.

*Sample analysis*

To prepare the solutions, firstly a 1M NaOH solution was prepared by dissolving 40g NaOH pellets in 1L water. To make the DTPA solution, 39.3mg DTPA was suspended in 5mL distilled water. With the contents being constantly stirred, 1M NaOH was added drop wise until the DTPA was completely dissolved. The final volume was made up to 10mL with distilled water. This solution was stable at room temperature and was prepared twice over the course of the study. Before each visit the NEM solution was prepared by dissolving 12.5mg NEM in 10mL of distilled water. This was kept at 4°C and fresh reagent was prepared for each study visit.

The required ethanol was first chilled to 0°C. 0.5mL of each sample was placed in a 1.5mL microcentrifuge tube and 1mL of cold ethanol was added. The tubes were then vortexed for 10 seconds and placed at 0°C for 30 minutes. Samples were centrifuged at 14,000rpm for five minutes. The supernatant was removed for the determination of nitrate and nitrite. Calibration of the device is ensured by a maintenance contract with Analytix and a service is provided twice per year. The internal calibration of the analysis is performed by using serial dilutions of nitrate and nitrite standards with known concentrations. In addition, each run of the samples is preceded by the injection of a standard with known concentration to ensure the accuracy of the results.

Briefly, prior to analysis a standard curve was created for both nitrate and nitrite and an equation was derived to calculate nitrate and nitrite concentrations by fitting a linear regression line to the standard concentrations. R^2^ was greater than 0.99. Diluted samples were injected into the purge vessel using a glass Hamilton (Fisher) syringe with an injection volume ranging from 10μL to 100μL. The injection volume was adjusted to maximise the sensitivity of the measurements and taken into account when calculating the final concentrations. The area under the curve of the peaks was calculated automatically by the Analytix software, which minimises the risk of between-operator differences in the calculation of results, and was used to calculate the concentrations of the samples using the standard regression equations. Samples were analysed in singlicate and quality of the peaks was checked visually based on height, fronting and tailing. Analyses were repeated for samples with low quality peaks. The assay performance of the nitrate and nitrite analysis is high, with a within-operator coefficient of variation of 91% for nitrate and 89% for nitrite after a series of ten consecutive manual injections of a standard solution with known concentration.
